# Ellis Jones technique as a cost-effective treatment for chronic peroneal tendon subluxation: A case report

**DOI:** 10.1097/MD.0000000000045139

**Published:** 2025-10-03

**Authors:** The Hung Hoang, Hoang Anh Dang, Tran Canh Tung Nguyen

**Affiliations:** a Department of Trauma and Orthopaedic Surgery, Military Hospital 103, Vietnam Military Medical University, Hanoi, Vietnam.

**Keywords:** case report, Ellis Jones technique, peroneal tendon subluxation

## Abstract

**Rationale:**

Peroneal tendon subluxation is an uncommon injury that is often overlooked during the acute phase, resulting in long-term instability.

**Patient concerns:**

We present a case involving a 25-year-old male soldier with a 2-year history of recurrent ankle pain and instability following an initial ankle injury.

**Diagnoses:**

Chronic peroneal tendon subluxation was diagnosed based on clinical examination and imaging findings.

**Interventions:**

Given the limitations of surgical instruments available at our institution in Vietnam, the patient was treated operatively using the Ellis Jones method, a traditional technique. The superior peroneal retinaculum was reinforced with a part of the Achilles tendon. Postoperatively, the leg was immobilized in a below-knee cast for 6 weeks.

**Outcomes:**

The patient experienced an uncomplicated recovery, returning to work without pain or need for braces within 2 months and resuming full physical activity 6 months postoperatively.

**Lessons:**

This case highlights that the Ellis Jones method remains a simple, cost-effective, and reliable surgical option for managing chronic peroneal tendon subluxation, particularly in resource-limited settings where specialized implants are unavailable.

## 1. Introduction

Peroneal tendon (PT) subluxation is a rare injury in ankle trauma that is most commonly seen in young people participating in strenuous activities. It is caused by sudden, forceful dorsiflexion of the foot along with a powerful contraction of the peroneal muscles, leading to a tear of the superficial peroneal retinaculum (SPR). Consequently, the PT can shift forward abnormally, resulting in ankle instability, persistent pain, and swelling.^[1,2]^ Although the exact prevalence of this injury remains unclear, research by Sammarco et al revealed that among 47 cases requiring surgery for ankle instability, nearly 30% showed PT tears caused by PT subluxation.^[3]^ Thus, raising awareness of this lesion among clinicians is necessary as it is easily missed during diagnosis, which leaves patients with chronic ankle pain and instability.^[4]^

Patients with chronic PT dislocation often report symptoms such as a history of popping or snapping sounds from the tendons, and the dislocated tendons may be visible, lying on top of the lateral malleolus. The tendons may dislocate freely upon activation of the peroneal muscles, particularly during dorsiflexion and eversion, and patients may be able to demonstrate this dislocation. If the dislocation is not obvious, patients may use their fingers to assist in identifying the dislocated tendons.^[5]^ Magnetic resonance imaging (MRI) is the most effective way to establish the diagnosis, as it can visualize both the dislocated tendon and the tear of SPR.^[6]^

Although most of the authors confirm that surgical intervention is the appropriate option for chronic PT dislocation, various surgical techniques have been described. These include repair of the SPR, bone block procedures, reinforcement of the SPR with local tissue transfers, rerouting of the tendons behind the calcaneofibular ligament, and groove-deepening procedures.^[7,8]^ Each technique has its own strengths and weaknesses. Surgeons should select the most appropriate approach based on patient characteristics and the resources available within their medical facility.

In our institution in Vietnam, we occasionally face shortages of specialized instruments and implants, especially for uncommon diseases. In this paper, we present a case of chronic PT dislocation that was surgically managed using SPR reconstruction with the Ellis Johns technique. This case report demonstrates the effectiveness of this classical surgical repair method and its applicability in settings where specialized instruments and implants are not readily available.

## 2. Case presentation

A 25-year-old male soldier presented with a 2-year history of left ankle injury in a dorsiflexion position, followed by swelling, difficulty walking, and pain in the lateral aspect of the ankle. He had been referred to physical therapy after being diagnosed with an ankle sprain but noted no improvement. Sometimes, the click sound was audible during the dorsiflexion with ankle inversion.

**Figure 1.**
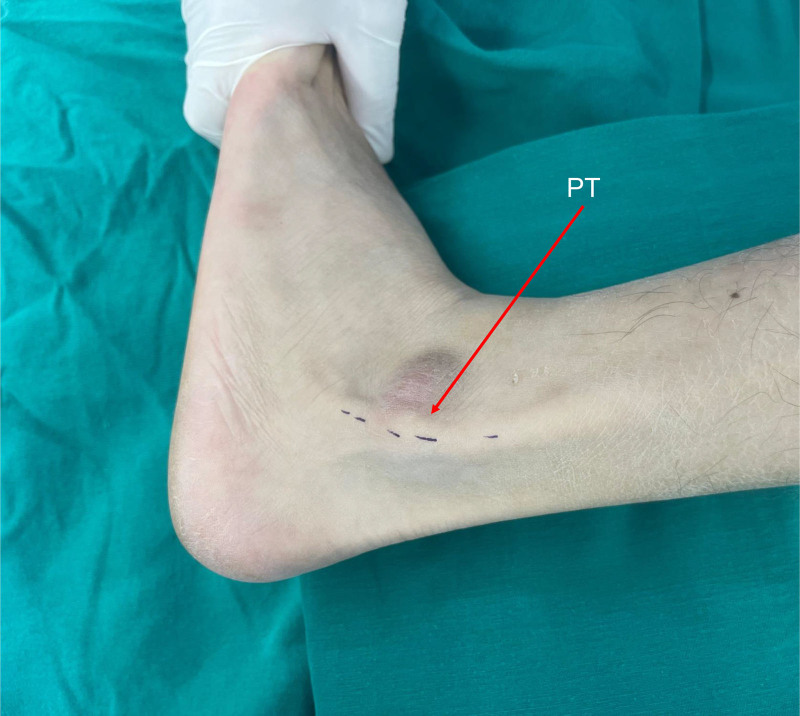
Clinical photograph showing dislocation of the peroneal tendon visible over the fibula. PT = peroneal tendon.

On admission, physical examination revealed a palpable prominence over the lateral malleolus, as well as tenderness to palpation along the peroneal tendons. A palpable click sensation was noted on resistance to eversion, suggestive of the PT dislocation, which was also visible on the fibula (Fig. 1). Neurological examinations, including sensation, motor function, and vascular assessment, were unremarkable. The preoperative American orthopedic foot and ankle society (AOFAS) score^[9]^ was 56 points (pain: 20 points, function: 26 points, and alignment: 10 points).

**Figure 2.**
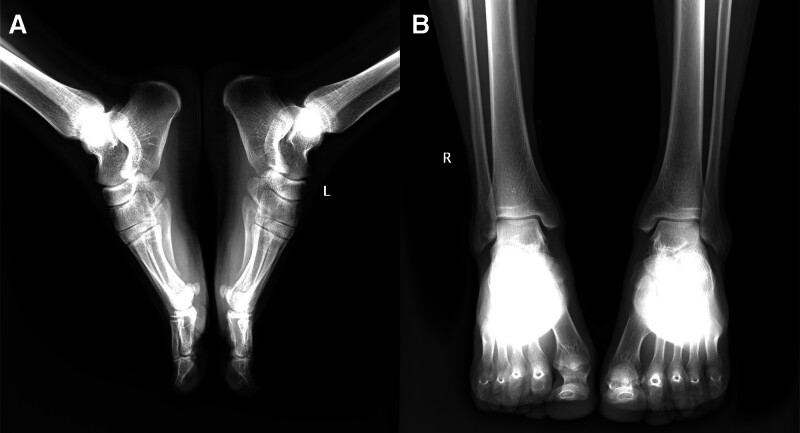
Preoperative ankle X-rays showing no fracture, dislocation, or other abnormalities. (A) Lateral view; (B) Anteroposterior view.

**Figure 3.**
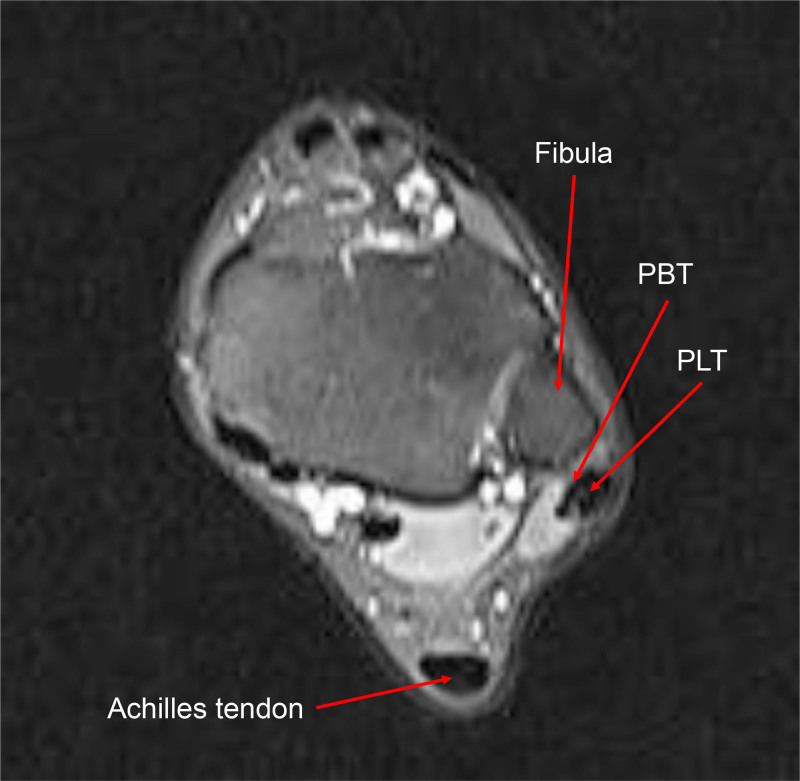
Preoperative MRI of the left ankle demonstrating peroneal tendon dislocation. The peroneus longus tendon, which normally lies posterolateral to the peroneus brevis tendon and posterior to the lateral malleolus, is laterally displaced over the fibula. MRI = magnetic resonance imaging, PBT = peroneus brevis tendon, PLT = peroneus longus tendon.

Preoperative radiographs of the ankle demonstrated no significant fractures, dislocations, or bone joint malalignment (Fig. 2). MRI showed PT dislocation with disruption of the SPR (Fig. 3).

The patient was positioned in the lateral decubitus position, and a tourniquet was applied to the upper thigh. An 8 cm incision was made along the posterior margin of the fibula, curving distally around the fibular tip in line with the excursion of the peroneal tendons and staying anterior to the Sural nerve. When SPR was exposed, it was found to be damaged due to long-term dislocation of PT and irreparable. Intraoperative provocative testing with ankle dorsiflexion and inversion had triggered subluxation and popping due to the peroneal retinaculum being ruptured.

**Figure 4.**
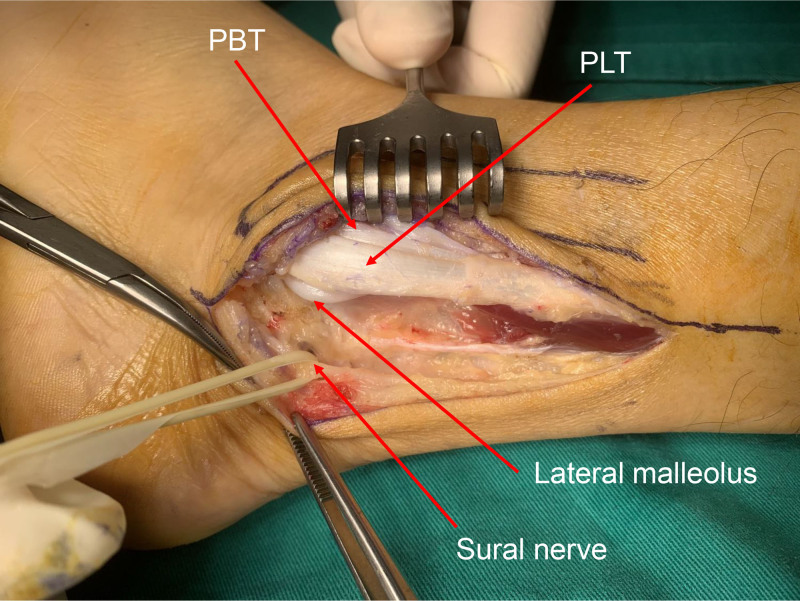
Intraoperative photograph showing the sural nerve separated by a string and the peroneal tendons exposed. PBT = peroneus brevis tendon, PLT = peroneus longus tendon.

**Figure 5.**
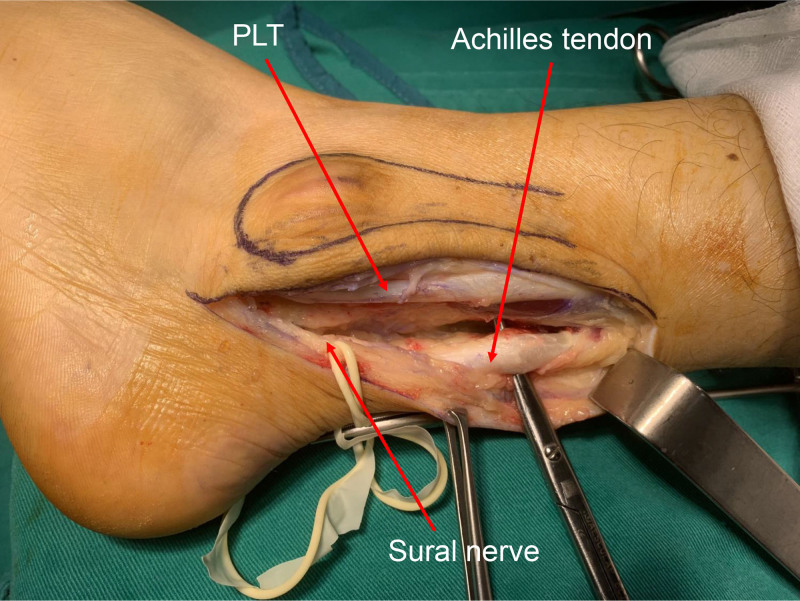
Intraoperative photograph showing exposure of the Achilles tendon. PLT = peroneus longus tendon.

**Figure 6.**
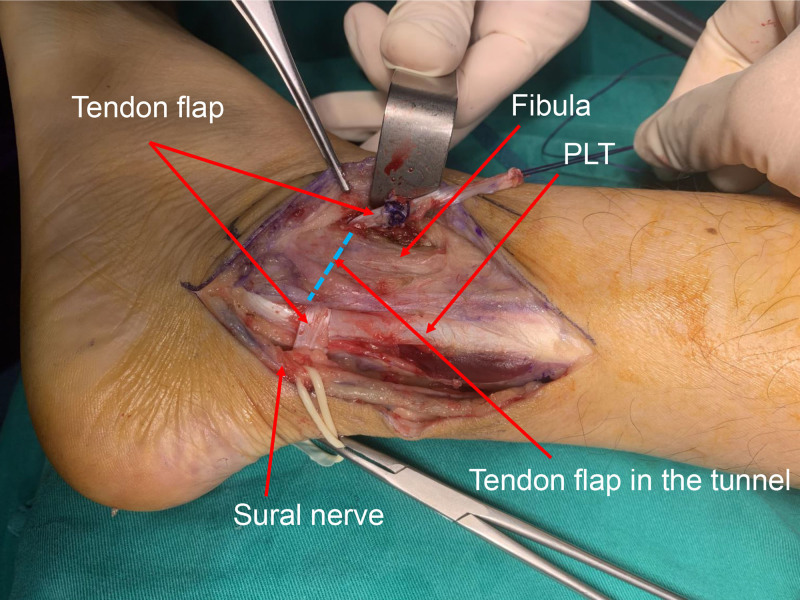
Intraoperative photograph showing the tendon flap drawn through the tunnel. The flap was positioned laterally to the peroneal tendons. The tunnel was drilled 2.5 cm above the tip of the lateral malleolus. PLT = peroneus longus tendon.

**Figure 7.**
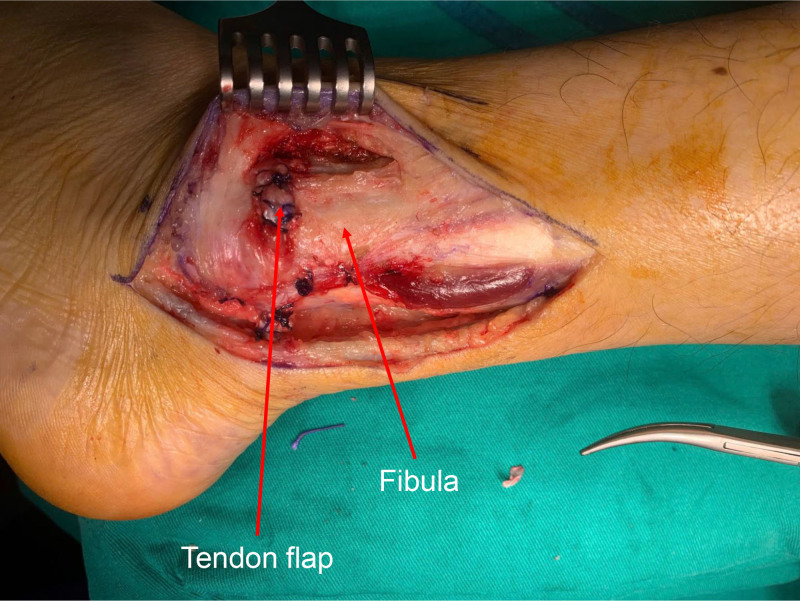
Intraoperative photograph showing the tendon flap sutured to itself and to the periosteum using 1–0 vicryl.

The Achilles tendon was exposed while avoiding damage to the Sural nerve and the accompanying short saphenous vein (Figs. 4 and 5). A lateral one-quarter segment of the Achilles tendon was harvested proximally, preserving its calcaneal insertion. The harvested tendon flap measured approximately 8 cm in length and 5 mm in diameter. The diameter of the tendon flap was 5 mm, and a drill bit was chosen equal to the diameter of the tendon flap. The lateral malleolus was exposed, and a tunnel, corresponding in diameter to the tendon flap, was created 2.5 cm above the tip of the lateral malleolus in an anteroposterior direction. The posterior aspect of the tunnel was positioned close to the fibular edge to avoid leaving a recess into which the tendons could slip. The free end of the flap from the calcaneum is drawn through the tunnel and sutured to itself and the periosteum using 1–0 vicryl sutures (Figs. 6 and 7). It is emphasized that the sutures should be tightened with the foot in dorsiflexion and supination to prevent postoperative limitation of dorsiflexion. After the reconstruction, the provocative ankle dorsiflexion with inversion was observed to no longer trigger the subluxation. The wound was irrigated, and layers were closed anatomically.

**Figure 8.**
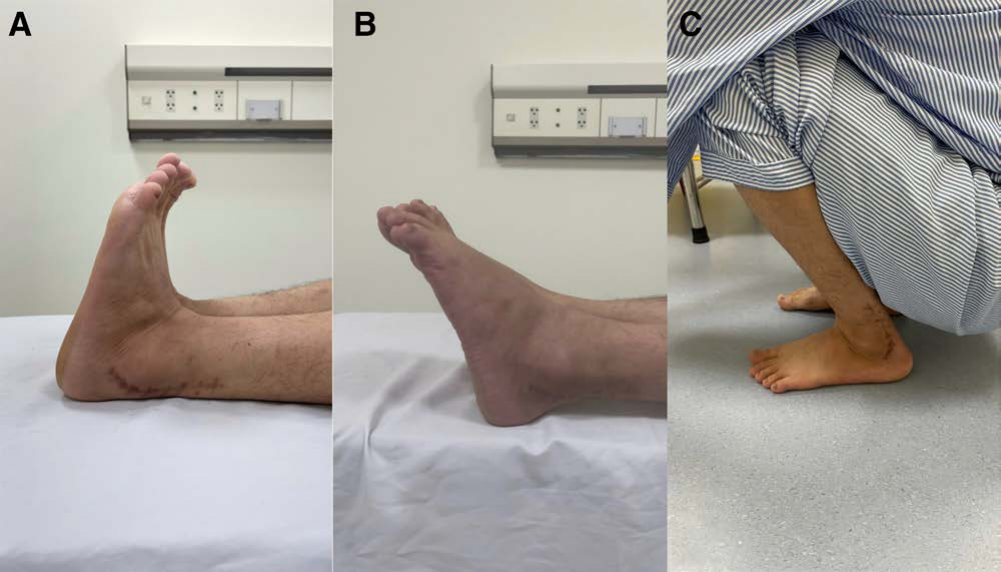
Six-month postoperative functional outcome. No limitation in range of motion and no recurrence of peroneal tendon subluxation were observed. (A) Dorsiflexion; (B) Plantarflexion; and (C) Full squat.

**Figure 9.**
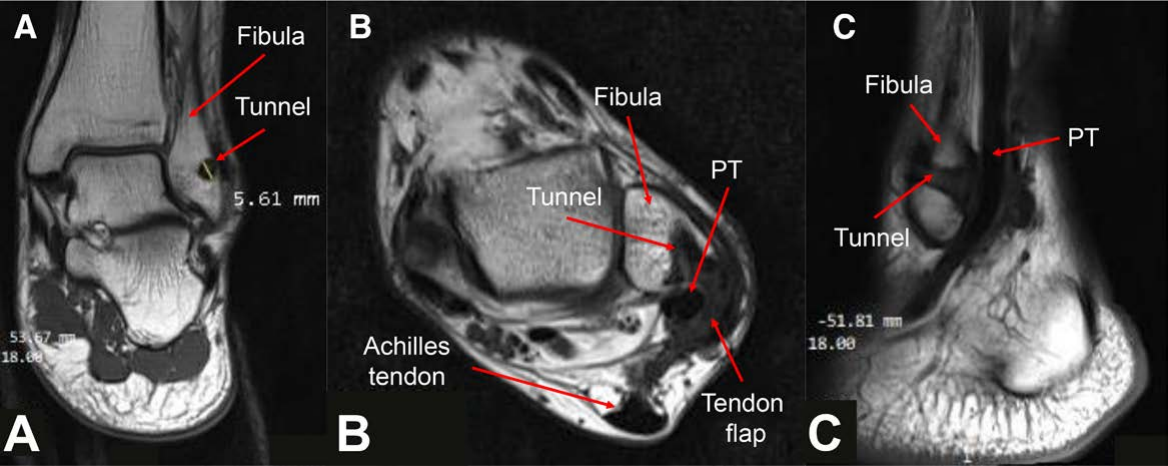
Postoperative MRI of the left ankle at 3-yr follow-up. (A) Coronal T1-weighted image demonstrating that the tendon flap has incorporated into the fibular tunnel; the tunnel diameter measures 5.61 mm. (B) Axial T1-weighted image showing the tendon flap incorporated into the fibular tunnel and the peroneal tendons in the correct anatomical position, without dislocation. (C) Sagittal T1-weighted image confirming the tendon flap incorporated into the fibular tunnel. MRI = magnetic resonance imaging, PT = peroneal tendon.

After the surgery, the leg was immobilized in a below-knee cast for 6 weeks, with the ankle in a neutral position. Following cast removal, the patient was allowed to put weight on the operated leg and exercise ankle movement. By 8 weeks postoperatively, the patient regained normal functionality without recurrence. Vigorous sports activities were restricted for an additional 6 months. At 6 months postoperatively, the patient demonstrated a full range of ankle motion on physical examination (Fig. 8). He was then transitioned to a walk-to-run program with physical therapy. The patient began work-ups for his duty, successfully transitioning to full duty without any restrictions. At twelve months postoperatively, the patient had returned to moderate-level sports activities, daily activities were not limited, ankle pain was absent, and good alignment was observed (AOFAS score: 93; pain: 40 points, function: 43 points, and alignment: 10 points). At 3 years postoperatively, the patient was completely free of ankle pain, able to engage in sports and strenuous military activities without limitation, and capable of marching 60 km over mountainous terrain while carrying a 20 kg backpack, with good alignment (AOFAS score: 100). MRI results showed no signs of recurrent tendon subluxation, and the tendon flap had healed into the bone tunnel (Fig. 9).

## 3. Discussion

Peroneal tendon dislocation is a rare condition. Espinosa et al (2015) reported that among more than 23,000 ankle injuries that occur every day in the United States, <0.5% were diagnosed as peroneal subluxations.^[10]^ The very low incidence and the same mechanism of ankle sprains may also be due to frequent misdiagnoses. In cases of chronic dislocation, the most important symptom is a palpable or audible clicking sensation posterior to the lateral malleolus. This occurs due to the displaced tendons snapping over the distal fibular head.^[5]^ Therefore, a comprehensive patient history and meticulous physical examination are crucial for accurate diagnosis. Additionally, patients need to have an MRI of the ankle since it is highly effective in identifying soft-tissue pathology, including SPR rupture, PT tears, tenosynovitis, and malposition of the tendons. Besides, radiographic imaging is also necessary to rule out bone and joint damage. A plain radiograph can reveal a grade 3 subluxation by visualization of an avulsed peace of the lateral malleolus. Computed tomography may further aid in evaluating the anatomy of the lateral malleolus, which is especially relevant for patients undergoing procedures such as bone block or groove-deepening techniques. Dynamic ultrasound has become useful to better assess subluxation than an MRI or computed tomography scan only, providing a positive predictive of 100% but is investigator-dependent.^[11,12]^

There have been various options reported for surgical repair of peroneal subluxation or dislocation. Each of these procedures has its strengths and weaknesses. Repair of the SPR is used most commonly for acute subluxation. The surgeon uses suture anchors to reattach the retinaculum onto the distal fibula after shaving down the insertion site. Excellent results and quick recovery time have been reported in the literature.^[13]^ Bone block procedures are technically demanding, require fixation with a screw, and are associated with long periods of bone healing. The groove-deepening procedure deepens the retromalleolar groove to provide a new, more stable bed for the peroneal tendon. However, it may cause an irregular bed for the tendons, with the possibility of adhesion formation, scarring, and subsequent restriction of motion. The risk of complications up to 30%.^[14]^ Different authors reported that the rerouting procedures had varying success rates. These were associated with high complication rates, including sural neuropathy and ankle instability. That is the reason why this method is not widely used.^[15,16]^ In the present case, the patient was an active-duty soldier with a history of regular training activity prior to injury, for whom rapid recovery after surgery and the ability to return to high-intensity military activities were critical. Therefore, in order to facilitate early return to duty and minimize complications, we did not select techniques involving bone block procedures or groove-deepening. In addition, the patient had suffered from chronic peroneal tendon dislocation for 2 years, which resulted in severe damage to the SPR, making primary repair difficult. At the same time, during the surgical period, medical facilities in Vietnam had limited access to modern surgical devices such as suture anchors, biocomposite screws, or adjustable-loop cortical suspensory fixation implants. After carefully weighing the advantages and disadvantages of available techniques, we selected the Ellis–Jones procedure for this patient.

The Ellis Jones technique, first described in 1932 for the treatment of chronic PT dislocation, involves reinforcement of the SPR using a pedicled graft from the Achilles tendon.^[17]^ Reconstruction using the Achilles tendon provides a robust and biologically compatible alternative for stabilizing the peroneal tendons or the calcaneofibular ligament. Compared to bone block and groove-deepening procedures, this method avoids complications such as delayed bone healing and the formation of irregular tendon beds. It is particularly well-suited for patients with prolonged subluxation prior to intervention. Also, several studies have shown that harvesting one-quarter to one-third of the Achilles tendon does not affect ankle function. The size of the Achilles tendon also returned to normal on MRI at 12 months postoperatively.^[18]^ Nayak et al^[19]^ reported favorable outcomes in 4 patients who underwent the Ellis Jones procedure for recurrent PT dislocations, with an average symptom duration of 7 months prior to surgery. Postoperative follow-up ranged from 2 to 9 years. All patients resumed athletic activities within an average of 8 months, with no reported complications, limitations in ankle range of motion, muscle atrophy, tendinitis, or Achilles tendon weakness. Similarly, Gökkuş et al^[20]^ described a case involving a patient with a 22-year history of chronic peroneal subluxation treated using the Ellis Jones technique. The patient returned to work within 8 weeks and was followed up for a period of 4 years after the operation, which revealed an excellent result. Postoperative MRI at the last visit revealed the healed synovitis.^[20]^ However, these authors primarily evaluated outcomes only based on the time to return to sports activities. Nayak et al assessed ankle function using a questionnaire addressing pain, ankle stability, physical activity, and postoperative complications, whereas Gökkuş et al did not use a standardized scoring system for ankle evaluation. In contrast, we assessed ankle function using the AOFAS score, which is a widely adopted tool for evaluating ankle outcomes after surgery. We hope that the use of a standardized scoring system to assess postoperative function will contribute additional data for future studies to enable comparison. In 2016, Van Dijk et al conducted a systematic review comparing surgical outcomes of chronic peroneal tendon dislocation between SPR repair alone and SPR repair combined with groove-deepening. The results showed no significant differences in ankle functional recovery according to the AOFAS score between the 2 methods. The preoperative and postoperative AOFAS scores in the SPR repair group were 67 ± 12 and 93 ± 9.79, respectively (*P* = .0249). In the SPR repair combined with groove-deepening group, the preoperative and postoperative AOFAS scores were 60 ± 5.6 and 94 ± 2.3, respectively (*P* = .0003).^[21]^

In the present case, the procedure involved SPR reinforcement using a flap of the Achilles tendon without groove-deepening. The patient was able to return to physical activity 6 months postoperatively. Compared to other techniques, this duration is similar. Various studies showed a duration between 2 and 6 months for patients to return to full sports activity.^[21,22]^ Maroc et al^[23]^ described 2 cases of chronic PT dislocation of 2- and 3-month durations, respectively, treated with simple SPR repair utilizing a periosteal flap from the lateral malleolus without groove-deepening. In contrast to our case, these patients required approximately 18 months post-surgery before resuming full sports activity. Furthermore, Deng et al^[22]^ conducted a comparative study evaluating clinical outcomes and return-to-sport intervals between patients undergoing SPR reattachment and those treated with bone block procedures. Both techniques demonstrated low rates of recurrent dislocation (8.33% for SPR reattachment vs 0.00% for bone block procedures); however, patients who underwent SPR reattachment returned to sports earlier, with a median recovery time of 5 months, compared to 6 months for those treated with bone block augmentation.

In situations where advanced surgical instruments or techniques are unavailable, the Ellis Jones method also has the advantage of economic efficacy since it does not utilize an implant or special support tools. In this patient, we used only 1 vicryl suture to secure the tendon graft, without the need for expensive modern devices such as anchors, a biocomposite screw, or an adjustable-loop cortical suspensory fixation implant. Although less expensive non-absorbable sutures could theoretically have been used, vicryl was selected because it is absorbable, widely available in our hospital, and provides adequate early tensile strength for tendon-to-bone healing. Importantly, its cost (approximately USD 5) was only about 1/60 to 1/160 of that of the aforementioned modern fixation devices, making it both practical and economical in our setting. Additionally, this method relies on the ability to heal the tendon to the bone tunnel and the tendon to itself, which will help patients to recover faster. Creating a secure tendon barrier creation also reduces the rate of recurrent dislocation. It emphasizes the importance of basic surgical skills and the ability to adapt to the available resources.

In conclusion, the management of chronic PT dislocation remains challenging, particularly in cases with prolonged symptom duration or irreparable retinacular structures. Through this case report, we aim to provide clinical awareness and help improve earlier diagnosis of this condition; we also demonstrate the effectiveness of surgical reattachment of the Ellis Jones method that our patients underwent. This method demonstrates favorable clinical outcomes, low recurrence rates, and facilitates an earlier return to activity, particularly in physically active patients. Additionally, its simplicity and cost-effectiveness make it especially valuable in settings with limited surgical resources. Therefore, even though the Ellis Jones technique was described nearly a century ago, it might still be applied in particular cases.

## Author contributions

**Conceptualization:** The Hung Hoang, Hoang Anh Dang.

**Data curation:** The Hung Hoang.

**Investigation:** The Hung Hoang, Tran Canh Tung Nguyen.

**Methodology:** The Hung Hoang, Tran Canh Tung Nguyen.

**Project administration:** Hoang Anh Dang.

**Resources:** Hoang Anh Dang.

**Supervision:** Hoang Anh Dang, Tran Canh Tung Nguyen.

**Validation:** Hoang Anh Dang, Tran Canh Tung Nguyen.

**Writing – original draft:** The Hung Hoang, Tran Canh Tung Nguyen.

**Writing – review & editing:** Hoang Anh Dang, Tran Canh Tung Nguyen.
